# Myeloid cell-derived coagulation tissue factor is associated with renal tubular damage in mice fed an adenine diet

**DOI:** 10.1038/s41598-021-91586-5

**Published:** 2021-06-09

**Authors:** Shu Yamakage, Yuji Oe, Emiko Sato, Koji Okamoto, Akiyo Sekimoto, Satoshi Kumakura, Hiroshi Sato, Mai Yoshida, Tasuku Nagasawa, Mariko Miyazaki, Sadayoshi Ito, Nigel Mackman, Nobuyuki Takahashi

**Affiliations:** 1 Division of Nephrology, Endocrinology, and Vascular Medicine, Tohoku University Graduate School of Medicine, Sendai, 980-8574 Japan; 2 Department of Community Medical Support, Tohoku Medical Megabank Organization, Tohoku University, Sendai, 980-8574 Japan; 3 Division of Clinical Pharmacology and Therapeutics, Tohoku University Graduate School of Pharmaceutical Sciences & Faculty of Pharmaceutical Sciences, Sendai, 980-8578 Japan; 4 Division of Hematology, Department of Medicine, UNC Blood Research Center, University of North Carolina at Chapel Hill, Chapel Hill, NC 29599-7520 USA; 5 Present Address: JR Sendai Hospital, Sendai, Japan; 6 Present Address: Katta Public General Hospital, Shiroishi, Japan

**Keywords:** Diseases, Nephrology, Pathogenesis

## Abstract

Patients with chronic kidney disease (CKD) commonly exhibit hypercoagulability. Increased levels of uremic toxins cause thrombogenicity by increasing tissue factor (TF) expression and activating the extrinsic coagulation cascade. TF is induced in monocytes and macrophages under pathological conditions, such as inflammatory diseases. However, the role of monocyte myeloid cell TF in CKD progression remains unclear. We aimed to clarify this issue, and the present study found that patients with CKD had elevated levels of D-dimer, a marker of fibrin degradation, which was associated with decreased estimated glomerular filtration rate and increased serum levels of uremic toxins, such as indoxyl sulfate. In vitro studies showed that several uremic toxins increased cellular TF levels in monocytic THP-1 cells. Mice with TF specifically deleted in myeloid cells were fed an adenine diet to cause uremic kidney injury. Myeloid TF deletion reduced tubular injury and pro-inflammatory gene expression in the kidneys of adenine-induced CKD but did not improve renal function as measured by plasma creatinine or blood urea nitrogen. Collectively, our findings suggest a novel concept of pathogenesis of coagulation-mediated kidney injury, in which elevated TF levels in monocytes under uremic conditions is partly involved in the development of CKD.

## Introduction

The number of patients with chronic kidney disease (CKD) has been increasing worldwide. Renal replacement therapies, such as hemodialysis and renal transplantation, are required to treat advanced stages of CKD^1,2^. CKD independently increases the risk of cardiovascular diseases and leads to a poor prognosis^3^. Information on the pathogenesis and therapeutic targets of this disease is required to improve the quality of life of patients.

Tissue factor (TF) is a membrane protein that regulates the extrinsic coagulation system^4^. In this system, TF forms a TF/FVIIa complex that activates FIX and FX to FIXa and FXa, respectively. FXa activates prothrombin to thrombin, which in turn polymerizes fibrinogen to form fibrin^4^. TF-dependent coagulation is linked to inflammation. Coagulation proteases located downstream of TF, including FVIIa, FXa, and thrombin, activate protease-activated receptors (PARs)^5^. The PAR family includes PAR-1 to PAR-4. Inflammation and fibrosis in various disease conditions are induced via signaling through PARs^5,6^.

Under basal conditions, TF is usually localized in extravascular cells, such as vascular smooth muscle cells (VSMCs), adventitial fibroblasts, and pericytes^7,8^. In contrast, TF expression is induced in vascular endothelial cells and monocytes/macrophages under pathological conditions that include cancer, sepsis, and inflammatory diseases^9^. Levels of TF antigen are increased in the plasma of hemodialyzed and CKD patients compared with control subjects^10,11^. In addition, TF antigen levels in CKD patients are inversely correlated with the glomerular filtration rate (GFR)^10^. CKD is characterized by an accumulation of uremic toxins, such as indoxyl sulfate (IS), which elevates TF levels in endothelial cells and peripheral blood mononuclear cells^10,12^.

The role of TF-dependent activation of coagulation in CKD progression has attracted considerable research interest. Notably, monocyte TF is involved in pro-coagulant activity and tissue injury in inflammatory diseases^13–15^ and monocyte-derived macrophages and related chemokines contribute to the development of CKD^16,17^. However, the pathological role of monocytic TF in CKD progression is still unclear. To address this issue, we demonstrated that uremic toxins increased TF expression in THP1 monocytes and that the deletion of myeloid-derived TF alleviated kidney injury induced by adenine.

## Materials and methods

### Human study

This study was approved by the institutional review board of Tohoku University (No. 2019–1-602). Written informed consent was obtained from all enrolled patients. This study was performed in accordance with the principles of the Declaration of Helsinki. Patients who underwent renal biopsy in the renal division of Tohoku University were enrolled in our renal biopsy cohort to compare their clinical and histological findings and metabolite profiles, including uremic toxins. Although the indications for renal biopsy were judged by each physician, CKD patients with urinary abnormality, nephrotic syndrome, rapid progressive renal insufficiency, or hereditary glomerulopathy were generally eligible^18^. The data from 72 such patients who underwent renal biopsy from July 2018 to June 2019 were analyzed in this study. Patients less than 20 years of age and those with acute kidney injury, rapidly progressive glomerulonephritis, acute tubulointerstitial nephritis, or representative primary nephrotic syndromes (minimal-change nephrotic syndrome, focal segmental glomerular sclerosis, and membranous nephropathy) were excluded. Patients with malignancy, aortic dissection, aortic aneurysm, or systemic amyloidosis as well as those undergoing steroid treatment were also excluded. Finally, 37 patients were included in the study. The general characteristics of the patients obtained from their medical records included age, sex, body mass index, blood pressure, laboratory findings, and renal biopsy findings. Plasma D-dimer levels were measured using a Nanopia D-dimer kit (Sekisui Medical, Tokyo, Japan). Serum samples were collected from patients on the first day of admission for further analyses of their metabolite profiles.

### Cell culture

The THP-1 human monocytic leukemia cell line was obtained from the Cell Resource Center for Biomedical Research at the Institute of Development, Aging, and Cancer at Tohoku University. The cells were grown in RPMI medium (Sigma-Aldrich, St. Louis, MO, USA) containing 10% fetal bovine serum, 1% L-glutamine, and 1% penicillin–streptomycin at 37 °C in a 5% CO_2_-humidified atmosphere. In the first experiment, THP-1 cells were incubated with 500 μM IS (Sigma-Aldrich), 50 μM indole-3-acetic acid (IAA; Wako Pure Chemical Industries, Osaka, Japan), 500 μM p-cresyl sulfate (PCS; Tokyo Chemical Industry, Tokyo, Japan), and 100 μM methylglyoxal (MG; Sigma-Aldrich) for 24 h. The concentrations of these uremic toxins were determined based on the serum concentrations of uremic patients or established doses used in culture studies^19–21^. U-0126 (10 μM, Wako Pure Chemical Industries, Osaka, Japan) and Bay 11-7082 (10 μM, Sigma-Aldrich) were administered 1 h before IS treatment. In the second experiment, THP1 cells were treated with several coagulation proteases, including FVIIa, FXa, and thrombin, at a concentration of 10 IU/mL for 4 h (Haematologic Technologies, Essex Junction, VT, USA). All procedures were performed 24 h after serum starvation.

### Animal study

All animal experiments were conducted in accordance with the guidelines of Tohoku University and the ARRIVE. The experimental protocol was approved by the Institutional Animal Care and Use Committee of Tohoku University. A C57BL/6J murine model with a deletion of the myeloid TF gene (*LysM*^*Cre*+^*; Tf*^*fl*^*°*^*x/fl*^*°*^*x*^) was previously generated and maintained at the animal facility of Tohoku University^14,15^. The expression of TF in myeloid cells was selectively reduced by 90% by crossbreeding the *Tf*^*fl*^*°*^*x/fl*^*°*^*x*^ mice with mice expressing Cre recombinase under the control of the lysozyme (LysM) promoter^14^. The male 10–14 week-old *LysM*^*Cre*+^*; T*^*ffl*^*°*^*x/fl*^*°*^*x*^ mice (hereafter referred to as *Tf*^*ΔMye*^) and their littermate controls (*LysM*^*Cre-*^*; Tf*^*fl*^*°*^*x/fl*^*°*^*x*^*,* referred to as *Tf*^+*/*+^) were housed at 24 °C under a 12:12 h light–dark cycle and fed water ad libitum. To induce uremic kidney injury, the mice were fed an adenine diet (0.2%; Oriental Yeast Co., Ltd., Tokyo, Japan) for 4 weeks. Plasma, urine, and kidneys were then harvested for further analyses.

### Measurement of blood urea nitrogen (BUN) and plasma creatinine levels

BUN levels were measured using a colorimetric detection kit (Arbor Assays, Ann Arbor, MI, USA). Plasma creatinine levels were measured using liquid chromatography-tandem mass spectrometry (LC–MS/MS), as described in our previous study^22^.

### Measurement of uremic toxin levels

Quantitative analysis of IS, PCS, and IAA levels using LC–MS/MS was performed as previously described^23^. A Nanospace SI-II HPLC platform (Shiseido, Tokyo, Japan) coupled to a TSQ Quantum Ultra mass spectrometer (Thermo Fisher Scientific, Waltham, MA, USA) operating in the negative mode was used for quantitative analysis. Each sample (3 mL) was injected onto a 150 × 2.0 mm YMC-Pack Pro C18 3-mm column (YMC, Kyoto, Japan) at a flow rate of 0.3 mL/min. In the gradient elution, mobile phase A was 10 mM ammonium acetate and mobile phase B was acetonitrile. Linear and stepwise gradients were programmed as follows: 0–1 min, 0%–10% solvent B; 1–2 min, 10%–20% solvent B; 2–3 min, 20%–80% solvent B; 3–5 min, 80%–100% solvent B; 5–7 min, 100% solvent B; and 7–10 min, 0% solvent B. Quantitative MS/MS analysis was performed by a select reaction monitoring mode in which the transitions of the precursor ion to the product ion and collision energy (eV) were monitored: m/z 212 → 80, 21 eV for IS; m/z 216 → 80, 30 eV for IS-d4; m/z 187 → 107, 23 eV for PCS; and m/z 176.2 → 130, 14 eV for IAA. The spray voltage was 2500 V, vaporizer temperature was 450 °C, and capillary temperature was 220 °C.

### Enzyme-linked immunosorbent assay (ELISA) of TF protein

To extract the total protein, THP-1 cells were lysed in RIPA buffer (Cell Signaling Technology, Inc., Danvers, MA, USA) containing 20 mM Tris–HCl, 150 mM NaCl, 1 mM Na_2_-EDTA, 1 mM EGTA, 1% NP-40, 1% sodium deoxycholate, 2.5 mM sodium pyrophosphate, 1 mM β-glycerophosphate, 1 mM Na_3_VO_4_, 1 µg/mL leupeptin, and a protease inhibitor cocktail (Sigma-Aldrich). The cell extracts were centrifuged at 10,000 rpm for 5 min at 4 °C. The supernatants were stored at − 80 °C until further use. The levels of human TF protein in THP-1 cells were measured using an ELISA kit according to the manufacturer’s instructions (#DCF300, R&D Systems, Minneapolis, MN, USA).

### Histological evaluation

Kidney samples were fixed in 2% paraformaldehyde (PFA). The fixed tissues were embedded in paraffin and cut into 3 μm-thick sections. The sections were stained with Masson–Goldner solution and hematoxylin and eosin. More than five consecutive fields of renal cortex were examined on each slide at 100 × magnification. To evaluate histological tubulointerstitial injury, we measured the ratio of the tubular area and tubular lumen area to the renal cortex area using Image J software version 1.49 (NIH, Bethesda, MD; https://imagej.nih.gov/ij) in a blinded manner.

### Immunohistochemistry (IHC)

The kidney samples were fixed in 2% PFA, embedded in paraffin, and cut into 3 µm-thick sections. The sections were treated to retrieve antigen using proteinase K (Dako, Glostrup, Denmark), followed by overnight incubation at 4 °C with rabbit anti-human CD68 (1:2000, Abcam, Cambridge, UK) and rabbit anti-human fibrin/fibrinogen (1:4000, Dako, Glostrup, Denmark) primary antibodies. N-Histofine simple stain kits (Nichirei Biosciences, Inc., Tokyo, Japan) were used as secondary antibodies. Sections not incubated with primary antibodies were used as the negative controls. Five fields in the cortical regions were randomly selected and imaged at 100 × magnification. The positive area per cortical region was quantified using ImageJ software.

### Quantitative reverse transcriptase polymerase chain reaction (qRT-PCR)

Total RNA was extracted from whole kidney samples or THP-1 cells using TRIzol reagent (Invitrogen, Carlsbad, CA, USA). Reverse transcription was performed using an iScript Advanced cDNA Synthesis kit (Bio-Rad, Hercules, CA, USA) according to the manufacturer’s instructions. The SsoAdvanced Universal Probe/SYBR Supermix kit (Bio-Rad) was used to perform qRT-PCR. Hypoxanthineguanine phosphoribosyltransferase (*Hprt*) or *β-actin* was used as the reference gene. The primers used have been previously reported^24,25^. Their sequences are shown in Supplementary Table 1.

### Statistical analyses

All analyses were performed using JMP Pro 15 (SAS Institute Inc., Cary, NC, USA). Statistical significance was set at *p* < 0.05. In the human study, the Shapiro–Wilk test was performed to test for normality. Student’s *t*-test or one-way analysis of variance (ANOVA) with the Tukey–Kramer test was used to compare parametric variables. Nonparametric variables were compared using the Mann–Whitney U test or Kruskal–Wallis test followed by the Steel–Dwass test. The χ^2^ test was used to compare the categorical variables. The results are expressed as the mean ± standard deviation (SD) and median with interquartile range for normally and non-normally distributed variables, respectively.

In the cell culture and animal studies, between-group analyses were performed using Student’s *t*-test or one-way ANOVA with the Tukey–Kramer test after assessing normality using the Shapiro–Wilk test. A 1.5 × interquartile range rule was utilized to detect outliers which were excluded from the qRT-PCR data in Figs. 3 and 6. The results are shown as the mean ± standard error of the mean (SEM).

## Results

### Elevated D-dimer levels are associated with renal dysfunction and increased serum uremic toxin levels in our renal biopsy cohort

Patients with CKD have an activated coagulation system^26,27^. Therefore, we assessed the associations among renal function, coagulation abnormalities, and uremic toxin levels in the patients in our renal biopsy cohort. The basal characteristics of the patients are presented in Table 1. Of the 37 patients, 19 had CKD stage 1 or 2 (estimated GFR [eGFR] ≥ 60) and 18 had CKD stage 3 or 4 (eGFR < 60). Common findings of renal biopsy included mesangial proliferative glomerulonephritis (IgA nephropathy, 37.8%) and nephrosclerosis (29.7%). Patients with CKD stages 3 and 4 were older and mostly male, with a high prevalence of hypertension and increased plasma fibrinogen and blood D-dimer levels compared to patients with CKD stages 1 and 2. Serum IS levels were significantly higher in patients with CKD stages 3 and 4 (2.19 μg/mL) than in patients with CKD stages 1 and 2 (0.79 μg/mL).

**Table 1:** Basal characteristics of the renal biopsy cohort.

	CKD stage			
	Total (n = 37)	CKD 1–2 (n = 19)	CKD 3–4 (n = 18)	*p*-value
Age (years)	49.1 ± 17.8	40.3 ± 14.9	58.4 ± 15.8	0.0011
Sex (male)	17 (45.9%)	4 (21.1%)	13 (72.2%)	0.0018
BMI (kg/m^2^)	25.0 ± 5.02	24.8 ± 5.84	25.3 ± 4.13	0.7570
Current smoking	9 (24.3%)	4 (21.1%)	5 (27.8%)	0.5631
**Past history**				
Hypertension	22 (59.4%)	8 (42.1%)	14 (77.8%)	0.0272
Diabetes	7 (18.9%)	2 (10.5%)	5 (27.8%)	0.1805
**Findings of renal biopsy**				
Nephrosclerosis	11 (29.7%)	5 (26.3%)	6 (33.3%)	0.2258
Diabetic nephropathy	3 (8.1%)	0 (0%)	3 (16.7%)	
Mes PGN	14 (37.8%)	8 (42.1%)	6 (33.3%)	
Others	9 (24.3%)	6 (31.6%)	3 (16.7%)	
**Chemistry**				
U-protein (g/gCre)	1.17 (0.44–2.89)	1.17 (0.41–3.18)	1.22 (0.43–2.84)	0.8197
Creatinine (mg/dL)	0.91 (0.69–1.58)	0.69 (0.64–0.74)	1.58 (1.24–1.89)	< 0.0001
eGFR (mL/min/1.73 m^2^)	64.0 (36.0–78.0)	76.0 (68.0–92.0)	36.0 (29.8–51.0)	< 0.0001
Total protein (g/dL)	6.78 ± 0.61	6.85 ± 0.64	6.70 ± 0.58	0.4530
Serum albumin (g/dL)	3.79 ± 0.55	3.92 ± 0.51	3.64 ± 0.56	0.1253
T-cho (mg/dL)	206.3 ± 41.2	209.0 ± 38.1	203.6 ± 45.1	0.7039
TG (mg/dL)	160.5 (105.8–287.8)	124.5 (81.0–338.8)	166.5 (130.5–260.8)	0.2750
**Coagulation**				
Fibrinogen (mg/dL)	311.3 ± 68.5	283.1 ± 59.5	342.8 ± 65.1	0.0071
D-dimer (μg/mL)				
< 0.5	8 (22%)	4 (21%)	4 (24%)	0.0121
0.5 ≤ < 1	22 (61%)	15 (79%)	7 (41%)	
1 ≤	6 (27%)	0 (0%)	6 (35%)	
**Uremic toxins**				
Indoxyl sulfate (μg/mL)	1.39 (0.58–3.27)	0.79 (0.53–1.66)	2.19 (1.34–5.37)	0.0041
Indole-3-acetic acid (μg/mL)	0.99 (0.90–1.11)	0.98 (0.82–1.02)	1.05 (0.95–1.21)	0.0635
p-cresyl sulfate (μg/mL)	1.54 (0.19–5.15)	1.01 (0.22–2.15)	2.76 (0.14–9.41)	0.1621

CKD, chronic kidney disease; BMI, body mass index; Mes PGN, mesangial proliferative glomerulonephritis; U-protein, urinary protein; gCre, g creatinine; Cre, creatinine; eGFR, estimated glomerular filtration rate; T-cho, total cholesterol; TG, triglyceride. There is one missing data point in the plasma fibrinogen and D-dimer data. The data are shown as the mean ± SD or as the median with interquartile range.

**Figure 1:**
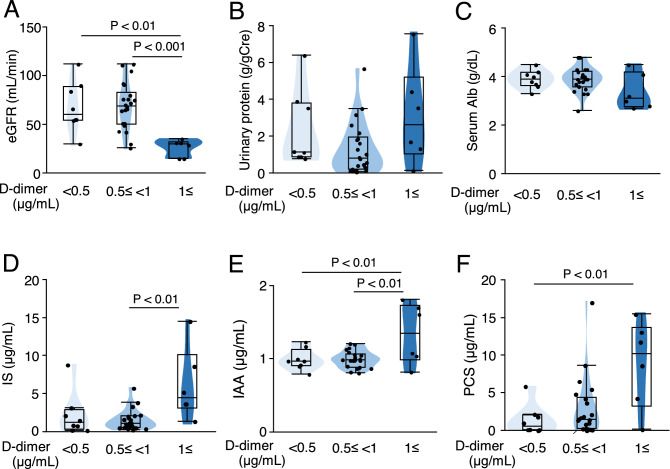
Association between plasma D-dimer and uremic toxin levels in CKD patients. Association between plasma D-dimer levels and (**A**) estimated glomerular filtration rate (eGFR), (**B**) urinary protein levels, (**C**) serum albumin (Alb) levels, (**D**) indoxyl sulfate (IS) levels, (**E**) indole-3-acetic acid (IAA) levels, and (**F**) p-cresyl sulfate (PCS) levels. gCre, g creatinine. n = 6–22. Data are shown as medians with interquartile ranges.

The D-dimer level, which is an indicator of fibrin degradation, was used as a coagulation parameter. To investigate the relationships among D-dimer level, renal function, and uremic toxin levels, the patients were classified into three groups (< 0.5, 0.5 ≤  < 1.0, and 1.0 ≤ μg/mL) based on standard cutoff values of D-dimer levels. Elevated D-dimer levels of ≥ 0.5 μg/mL and ≥ 1.0 μg/mL were observed in 28 patients (88%) and 6 patients (27%), respectively. Patients with D-dimer levels ≥ 1.0 μg/mL had lower eGFR and higher serum levels of IS, IAA, and PCS than those in the other groups (Fig. 1A–F).

### IS, IAA, and MG increase TF expression in THP-1 monocytic leukemia cells

**Figure 2:**
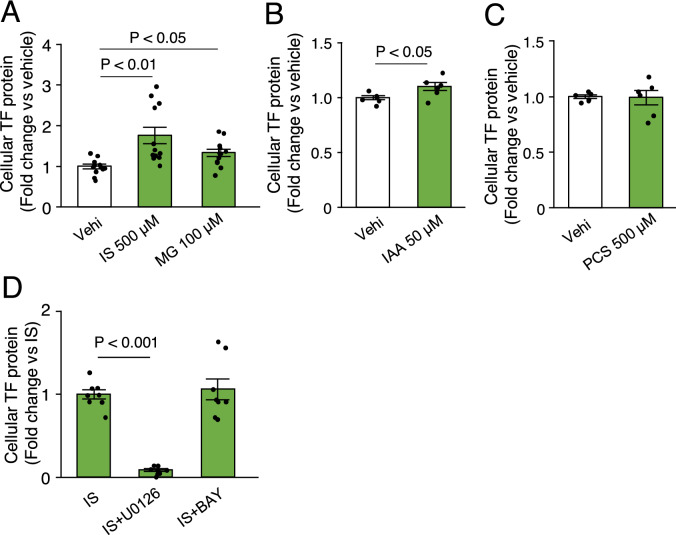
Effect of uremic toxins on TF expression in monocytic THP-1 cells. (**A–B**). Treatment with indoxyl sulfate (IS), methylglyoxal (MG), and indole-3-acetic acid (IAA) for 24 h increased the expression of TF protein in THP-1 cells. (**C**) Treatment with p-cresyl sulfate (PCS) did not affect the expression of TF protein in THP-1 cells. (**D**) Elevation of TF protein levels induced by IS was reduced by U-0126, but not by Bay 11–7082 (BAY). n = 6–8. vehi, vehicle. Data are shown as the mean ± SEM.

Previous studies have reported a link between uremic toxins and thrombogenicity in uremia^10,12,28^. Therefore, we evaluated whether uremic toxins (e.g., IS, IAA, PCS, and MG) could increase the expression of TF, a regulator of the extrinsic coagulation cascade, in THP-1 monocytes. Treatment of THP-1 cells with 500 μM IS and 100 μM MG for 24 h significantly elevated the levels of cellular TF proteins. Similarly, 50 μM IAA increased the levels of cellular TF proteins in THP-1 cells (Fig. 2A,B). In contrast, 500 μM PCS did not affect cellular TF levels in THP-1 cells (Fig. 2C). Given that the intracellular signaling, which regulates TF expression, is diverse^29^, we next explored the roles of mitogen-activated protein kinase (MAPK) and nuclear factor-kappa B (NF-κB) signaling in IS-mediated TF expression. U-0126, an inhibitor of MAPK signaling, significantly reduced the increase in TF levels induced by IS. In contrast, Bay 11-7082, an inhibitor of NF-κB signaling, had no effect on TF levels (Fig. 2D).

### Effect of coagulation proteases on the expression of inflammatory genes in THP-1 cells

**Figure 3:**
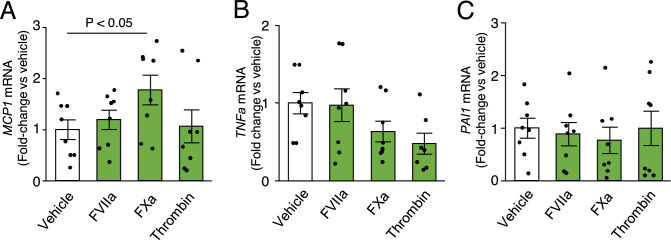
Effects of coagulation proteases on pro-inflammatory gene expression in THP-1 cells. (**A**) *MCP1*, (**B**) *TNFα*, and (**C**) *PAI1* mRNA levels in monocytic THP-1 cells treated with vehicle, FVIIa, FXa, and thrombin at 10 IU/mL. n = 7–8.

Activated coagulation proteases, such as FVIIa, FXa, and thrombin, are located downstream of TF. They increase inflammation^5^ and are likely involved in the pathogenesis of CKD. Pro-inflammatory molecules related to tumor necrosis factor-alpha (TNFα) signaling, the monocyte chemoattractant protein 1/chemokine (C–C motif) ligand 2 (MCP1/CCR2) pathway, or plasminogen activator inhibitor-1 (PAI1) expression reportedly contribute to CKD progression^30,31^. Therefore, we examined the effect of coagulation proteases on inflammatory gene expression in monocytic THP-1 cells. Examination of FVIIa, FXa, and thrombin (all at 10 IU/mL) revealed that FXa significantly increased *MCP1* mRNA levels (Fig. 3).

### Myeloid cell-specific TF deficiency reduces tubulointerstitial injury in mice fed an adenine diet

**Figure 4:**
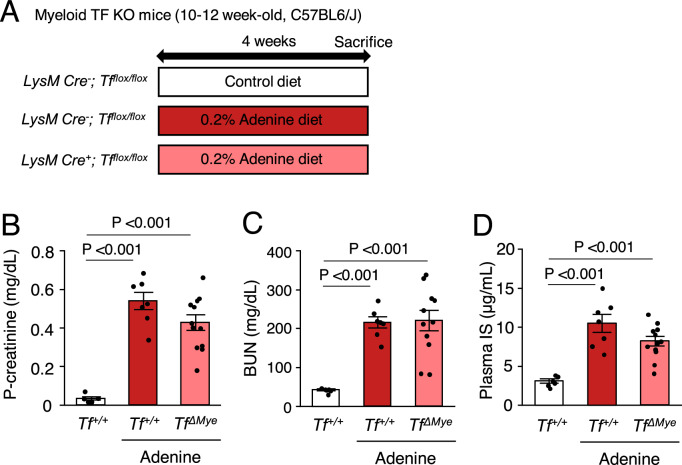
Levels of plasma creatinine, BUN, and IS in adenine-induced nephrotoxicity. (**A**) An experimental protocol for the diet administered to the animals in this study. TF, tissue factor. KO, knockout. (**B–D**) Comparison of the plasma creatinine (P-creatinine), blood urea nitrogen (BUN), and plasma indoxyl sulfate (IS) levels among groups. n = 6–12. Data are shown as the mean ± SEM.

**Figure 5:**
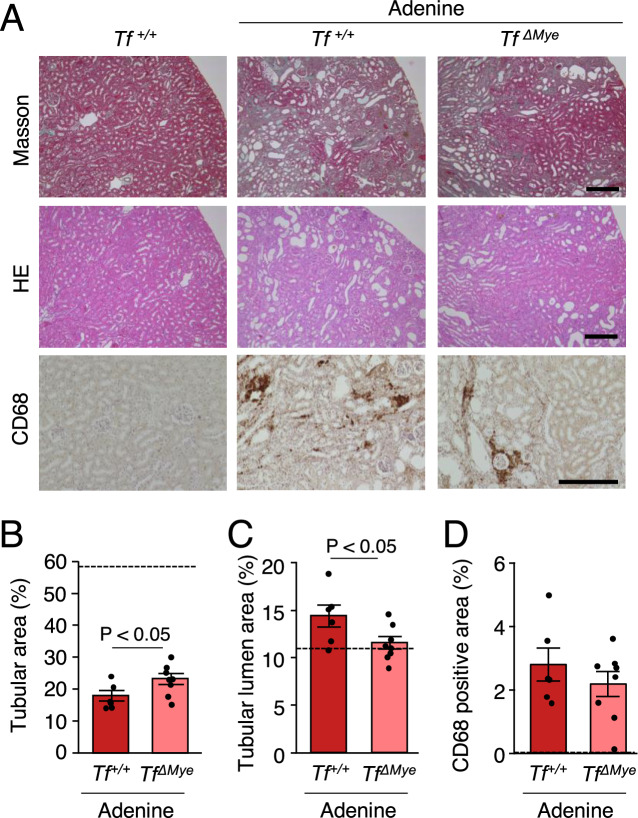
Myeloid TF deficiency alleviates renal tubular injury in mice fed an adenine diet. (**A**) Representative photomicrographs of Masson staining, hematoxylin and eosin (H&E) staining, and immunohistochemical staining of CD68. Comparison of the tubular area (**B**), tubular lumen area (**C**), and CD68-positive area (**D**) between *Tf*^+*/*+^ and *Tf*^*ΔMye*^ mice fed an adenine diet. Scale bar = 200 μm. Dotted line indicates the level of *Tf*^+*/*+^ fed normal chow. n = 6–8. Data are shown as the mean ± SEM.

Uremic conditions likely increase monocyte TF levels. However, whether monocyte TF is involving in CKD development is unknown. We examined the role of myeloid TF in an adenine-induced CKD mouse model. *TF*^+*/*+^ or *Tf*^*ΔMye*^ mice were fed an adenine diet (0.2%) for 4 weeks (Fig. 4A). Plasma levels of creatinine, BUN, and uremic toxins, such as IS, were markedly increased in mice fed an adenine diet compared to the mice fed normal chow. However, deletion of myeloid TF did not affect their plasma levels (Fig. 4B–D). Furthermore, plasma levels of PCS and IAA were unchanged by myeloid TF deletion in our model (data not shown). Reduced tubular area and increased tubular lumen dilatation are hallmarks of adenine-induced kidney injury^32^. *Tf*^*ΔMye*^ mice fed an adenine diet showed increased tubular area compared with *Tf*^+*/*+^ mice. The dilated tubular lumen area was significantly reduced in *Tf*^*ΔMye*^ mice compared with *Tf*^+*/*+^ mice, suggesting the improvement of tubular atrophy following the deletion of myeloid TF (Figs. 5A–C). In contrast, the positive area of CD68, a marker of macrophages, in the renal cortex was similar between the groups (Fig. 5D).

### Myeloid cell-specific TF deficiency ameliorates inflammation and TF levels in adenine-induced kidney injury

**Figure 6:**
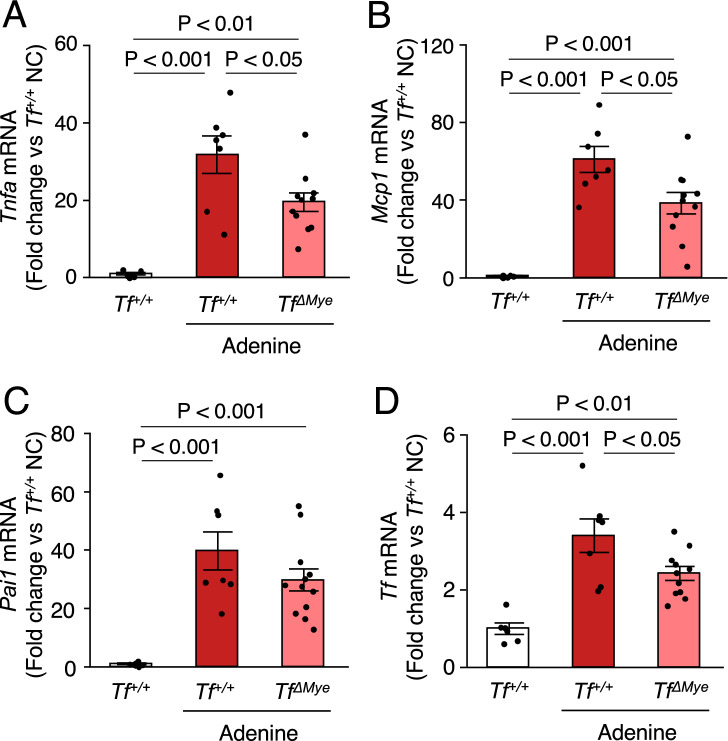
Myeloid TF deficiency reduces pro-inflammatory gene expression in adenine-induced tubular injury. (**A–D**) Comparison of *Tnfα*, *Ccl2*, *Pai1*, and *Tf* mRNA levels among groups. Data are shown as fold-change compared with *Tf*^+*/*+^ mice fed normal chow (NC). A.U., arbitrary unit. n = 7–12. Data are shown as the mean ± SEM.

To test whether histological injury in *Tf*^*ΔMye*^ mice is associated with a reduction in inflammation, we measured the expression of pro-inflammatory cytokines in the kidneys. *Tnfα* and *Mcp1* mRNA levels were significantly reduced by myeloid TF deletion in adenine-induced kidney injury mice. There were no changes in *Pai1* mRNA levels (Figs. 6A–C). *Tf* mRNA levels were significantly reduced by myeloid TF deletion (Fig. 6D).

### Fibrin deposition in adenine-induced kidney injury

Finally, we evaluated the effect of myeloid TF on fibrin deposition in the injured kidneys. The IHC-positive area of fibrin/fibrinogen was increased in adenine-induced kidney injury. However, the positive area was unchanged by the deletion of myeloid TF (Supplementary Fig. 1A-B).

## Discussion

In this study, we demonstrated that the levels of plasma D-dimer, a marker of coagulation activity, were negatively correlated with eGFR and positively correlated with plasma uremic toxins, such as IS, IAA, and PCS. Of the uremic toxins, IS, IAA, and MG increased TF protein levels in THP1 monocytic cells. FXa, a coagulation protease, increased *MCP1* mRNA levels in monocytic THP1 cells. Finally, myeloid-specific deletion of TF in mice with CKD and uremia induced by adenine alleviated histological damage and inflammation in their kidneys.

Increased levels of D-dimer were associated with elevated levels of IS, IAA, and PCS, suggesting a link between pro-thrombotic capacity and uremia. Similar to our observations, an increasing number of studies have highlighted the relationships among CKD, TF expression, and levels of uremic toxins. For example, IS and IAA levels were correlated with circulating TF or TF pro-coagulant activity in CKD patients^10,33^. An association between IS and several hemostatic factors, such as TF, von Willebrand factor, and soluble urokinase-type plasminogen activator receptor, has been reported^34^. Interestingly, increased IS level is also associated with an increase in the levels of an oxidative stress marker and neopterin, which indicates monocyte activation in the CKD cohort^34^. These findings suggest the involvement of IS in hemostatic system disruptions that are associated with an activation of monocytes under CKD pathogenesis.

The causal link between uremic toxins and pro-thrombotic reactions in CKD has been demonstrated in previous studies^10,12,28^. The relationship between indolic uremic solutes and TF activity is well described. IS and IAA increase the expression of TF in human umbilical vein endothelial cells, EA hy926 cells (a human endothelial cell line), and human peripheral blood mononuclear cells^10,12^. Indolic uremic solutes are potent ligands of the transcriptional factor aryl hydrocarbon receptor (AhR). Activated AhR translocates to the nucleus, regulating the expression of its target genes. It is demonstrated that the elevation of TF levels following stimulation of IS and IAA was inhibited by small interfering RNA or pharmacological inhibition of AhR in human endothelial cells or peripheral blood mononuclear cells^10^. The involvement of the NF-κB pathway in IAA-dependent upregulation of TF expression has been demonstrated in human endothelial cells^35^. The IS-AhR pathway reduces the interaction between TF and the ubiquitin ligase, STIP1 homology and U-box-containing protein 1 (STUB1), resulting in the inhibition of TF ubiquitination and an increase in its stability in VSMCs^36^. Our findings that IS and IAA can increase the expression of TF protein in THP-1 monocytic cells, and that MAPK is involving in the change, improve our understanding of thrombogenicity driven by indolic uremic solutes.

MG is a precursor of advanced glycation end products. The levels of these products are elevated in blood from patients with progressive CKD^37^. The role of MG in thrombogenicity has been addressed in several previous studies. One study reported that MG inhibits antithrombin activity that inhibits both thrombin and FXa expression^38^. Other studies have reported a negative correlation between platelet aggregability and thrombus stability^39^. These findings implicate MG as a novel regulator of uremic thrombogenicity in CKD patients. Our identification of the pro-coagulant capacity of MG by increasing TF protein levels in THP1 cells supports this regulatory role of MG.

We also observed a pro-inflammatory role of monocyte-derived TF in adenine-induced kidney injury. Deletion of myeloid TF reduced tubular injury scores and renal *Tnfα* and *Mcp1* mRNA levels. Monocyte TF can activate the coagulation cascade in inflammatory diseases^13,14^. Coagulation proteases downstream of TF exacerbate inflammation through PARs. In addition to our observation that FXa induced MCP1 expression in THP-1 cells, previous studies have demonstrated the pro-inflammatory roles of FVIIa, FXa, thrombin, and PAR agonist in monocytes and macrophages^40–42^. Furthermore, an in vivo study involving a kidney disease model demonstrated that pharmacological inhibition of FXa by edoxaban attenuated tubulointerstitial fibrosis, macrophage infiltration, and inflammatory molecule upregulation in unilateral ureteral obstruction^43^. Similarly, we demonstrated the therapeutic effect of FXa inhibitor on diabetic nephropathy^25^. PARs are highly expressed in the kidney cells, including tubular cells^44–46^. Deficiency of PAR2 or PAR1 reportedly alleviates renal fibrosis and inflammation in the models of tubular kidney injury^47–49^.

We observed a modest decrease in renal TF expression following the deletion of myeloid TF, which was accompanied by no or mild effects on renal function, inflammatory cell infiltration, or fibrin deposition in adenine-induced kidney injury. Based on our previous^49^ and unpublished preliminary data demonstrating the pronounced elevation of TF levels in kidney tubular cells in cisplatin-or adenine-induced kidney injury, one explanation for these modest effects on disease phenotypes is that other types of cells such as renal tubular cells may contribute to an increase in TF levels, renal coagulation, and inflammation in adenine-induced kidney injury. We plan to elucidate the role of TF in other types of cells in future studies.

This study has a few limitations. First, in our human study, a causal link between D-dimer and uremic toxin levels could not be determined because of its cross-sectional design. Additionally, a small number of patients were studied. Thus, a large-scale prospective study is required in the future. Secondly, human data on myeloid TF expression were not available in our cohort. But others demonstrated using flow cytometry that the increase of monocyte TF in patients with chronic renal failure or during hemodialysis was associated with other coagulation parameters^50^; myeloid TF under uremia is possibly important in coagulation status in CKD patients.

In conclusion, we found that uremic conditions increase the expression of TF in monocytes and that monocytic TF is partly involved in the pathogenesis of adenine-induced tubulointerstitial injury. These findings suggest a novel mechanism underlying the pathogenesis of coagulation-mediated kidney injury.

## Supplementary Information

Supplementary Information.
