# Supplementary material for: Myeloid cell-derived coagulation tissue factor is associated with renal tubular damage in mice fed an adenine diet
Source: Sci Rep. 2021 Jun 9;11:12159. doi: 10.1038/s41598-021-91586-5 (PMC8190319; doi:10.1038/s41598-021-91586-5)
Supplement: Supplementary file 1 — Supplementary Information. [file 41598_2021_91586_MOESM1_ESM.docx]

**Supporting information**

**Myeloid cell-derived coagulation tissue factor is associated with renal tubular damage in mice fed an adenine diet**

Shu Yamakage^1,a^, Yuji Oe^1,2,a^, Emiko Sato^1,3^, Koji Okamoto^1^, Akiyo Sekimoto^3^, Satoshi Kumakura^1^, Hiroshi Sato^1,3,#^, Mai Yoshida^1^, Tasuku Nagasawa^1^, Mariko Miyazaki^1^, Sadayoshi Ito^1,♭^, Nigel Mackman^4^, and Nobuyuki Takahashi^1,3^

^1^ Division of Nephrology, Endocrinology, and Vascular Medicine, Tohoku University Graduate School of Medicine, Sendai 980-8574, Japan.

^2^ Department of Community Medical Support, Tohoku Medical Megabank Organization, Tohoku University, Sendai 980-8574, Japan.

^3^ Division of Clinical Pharmacology and Therapeutics, Tohoku University Graduate School of Pharmaceutical Sciences & Faculty of Pharmaceutical Sciences, Sendai 980-8578, Japan.

^4^ Division of Hematology, Department of Medicine, UNC Blood Research Center, University of North Carolina at Chapel Hill, Chapel Hill NC, 29599-7520, USA.

# Current address is JR Sendai Hospital.

♭ Current address is Katta Public General Hospital.

**Supplementary Table 1.** Primer sequences

| Gene | Type | Sequence (5’-3’) |
| --- | --- | --- |
| SYBR green  Human  *ACTB*  *MCP1*  *TNFa*  *PAI1*  Mouse  *Pai1* | Forward  Reverse  Forward  Reverse  Forward  Reverse  Forward | TAKARA BIO (Shiga, Japan): #HA06780  ATAGCAGCCACCTTCATTCC  ATCCTGAACCCACTTCTGCT  TCTTCTCGAACCCCGAGTGA  CCTCTGATGGCACCACCAG  TGAGATCAGCACCACAGAC  ATTGATGATGAATCTGGCTCTC  TTCAGTGGCCAATGGAAGACTCCT |
| Taqman probe  Mouse  *Hprt*  *Tnfa*  *Mcp1*  *Tf* | Reverse  Forward  Reverse  Probe  Forward  Reverse  Probe  Forward  Reverse  Probe  Forward  Reverse  Probe | AGGGCAGTTCCACAACGTCATAC  GGACTGATTATGGACAGGAC  CAGAGGGCCACAATGTGAT  CCTCCCATCTCCTTCATGACATCTCG  CATCTTCTCAAAATTCGAGTGACAA  TGGGAGTAGACAAGGTACAACCC  CAGGTCGTAGCAAACCACCAAGTGGA  CTGGAGCATCCACGTGTTG  TGGGATCATCTTGCTGGTGA  AGCCAGATGCAGTTAACGCCCCACT  GAAAACTAACCAAAATAGCCCAG  TGATGAGTGTTTCTCCCAGG  TCCATTGCTCGGTGCACACTGTACTG |

**Supplementary Figure 1.** Fibrin deposition in adenine-induced tubular injury.

**A**. Representative photomicrographs of immunohistochemical staining against fibrin/fibrinogen. **B.** Comparison of the fibrin/fibrinogen-positive area. Dotted line indicates the level of *Tf^+/+^* fed normal chow. Scale bar = 500 μm. n = 6–8. Data are shown as the mean ± SEM.
